# Dietary obesity in mice is associated with lipid deposition and metabolic shifts in the lungs sharing features with the liver

**DOI:** 10.1038/s41598-021-88097-8

**Published:** 2021-04-22

**Authors:** G. J. P. Rautureau, B. Morio, S. Guibert, C. Lefevre, J. Perrier, A. Alves, M. A. Chauvin, C. Pinteur, M. A. Monet, M. Godet, A. M. Madec, J. Rieusset, A. Mey, Baptiste Panthu

**Affiliations:** 1 Univ Lyon, Centre de Résonance Magnétique Nucléaire à Très Hauts Champs (CRMN FRE 2034 CNRS, UCBL, ENS Lyon), 69100 Villeurbanne, France; 2 Univ Lyon, CarMeN Laboratory, INSERM, INRA, INSA Lyon, Université Claude Bernard Lyon 1, 69921 Oullins Cedex, France; 3 Hospices Civils de Lyon, Faculté de Médecine, Hôpital Lyon Sud, 69921 Oullins Cedex, France; 4 INSERM U.1060-CARMEN, Hôpital Lyon Sud Secteur 2, Bâtiment CENS ELI-2D, 165 Chemin du grand Revoyet, 69310 Pierre-Bénite, France

**Keywords:** Biomarkers, Bioenergetics

## Abstract

Obesity is associated with both chronic and acute respiratory illnesses, such as asthma, chronic obstructive pulmonary disease (COPD) or increased susceptibility to infectious diseases. Anatomical but also systemic and local metabolic alterations are proposed contributors to the pathophysiology of lung diseases in the context of obesity. To bring perspective to this discussion, we used NMR to compare the obesity-associated metabolomic profiles of the lung with those of the liver, heart, skeletal muscles, kidneys, brain and serum from male C57Bl/6J mice fed with a high-fat and high-sucrose (HFHSD) diet vs. standard (SD) chow for 14 weeks. Our results showed that the lung was the second most affected organ after the liver, and that the two organs shared reduced one-carbon (1C) metabolism and increased lipid accumulation. Altered 1C metabolism was found in all organs and in the serum, but serine levels were increased only in the lung of HFHSD compared to SD. Lastly, tricarboxylic acid (TCA)-derived metabolites were specifically and oppositely regulated in the serum and kidneys but not in other organs. Collectively, our data highlighted that HFHSD induced specific metabolic changes in all organs, the lung being the second most affected organ, the main alterations affecting metabolite concentrations of the 1C pathway and, to a minor extend, TCA. The absolute metabolite quantification performed in this study reveals some metabolic specificities affecting both the liver and the lung, that may reveal common metabolic determinants to the ongoing pathological process.

## Introduction

Obesity has reached epidemic proportions worldwide and affects the metabolism of all organs, impacting therefore whole-body homeostasis. As a consequence, obesity increases the risk of important chronic pathologies such as type 2 diabetes and cardiovascular diseases^1^. Whereas the liver, skeletal muscle, adipose tissue and the heart are classically studied in the context of obesity, others required more attention, such as the lung. Indeed, epidemiological studies have long shown that obesity is associated with both chronic and acute respiratory complications such as asthma, infectious diseases or chronic obstructive pulmonary disease (COPD)^2–6^. Therefore, the increased prevalence of obesity and the concomitant lung diseases has raised the need for a precise examination of obesity-induced alterations in lung metabolism^7^. This topic has gained outstanding relevance with the Covid-19 (SARS-CoV-2) pandemic and its severe pulmonary complications that particularly affect obese patients^8^.

The association between obesity and lung illnesses has long been discussed as the mechanical consequence of obesity on lung physiology and the systemic inflammation induced by dysfunctional adipose tissue^9^. Recent studies evidenced general and lung-localized metabolic alterations that may contribute to the pathophysiology of lung diseases in obesity. Notably, alterations of the lipid metabolism and the secretion of cytokines, or changes of the arginine metabolism leading to nitric oxide synthesis, have been proposed^2–4,10–12^. At the cellular level, impaired mitochondrial metabolism has also been observed^13,14^, and stressed mitochondria have been reported in the airway epithelial cells of high-fat and high-fructose fed mice^15^.

To further explore and characterize the array of specific alterations in lung metabolism induced by obesity, we used proton nuclear magnetic resonance (NMR) spectroscopy to analyze the metabolic profile induced in mice by an obesogenic diet, and we compared the metabolomes of lung to that of serum and five other tissues (liver, heart, skeletal muscle, kidneys and brain). Our NMR study enabled the quantification of 44 metabolites in organs and 32 metabolites in serum. It evidenced that the lung is one of the most metabolically affected organs, sharing specificities with the liver, and revealed unexpected organ-specific alterations of the one-carbon (1C) and tricarboxylic acid (TCA) pathways.

## Results

### HFHSD-induced obesity in mice

Metabolomic studies were performed in male C57Bl/6J mice fed either with a standard diet (SD) or a high-fat and high sucrose diet (HFHSD) for 14 weeks, a classical nutritional model of obesity^16^. As expected, HFHSD mice gained 65% more weight than SD animals (p < 0.0001, n = 12 per group) with a 57% increase of the weight of the liver (p < 0.0001) but not of the lung (Table 1). HFHSD mice showed significant hyperglycemia compared to SD mice (p < 0.0001, Table 1). As shown on Supplementary Fig. 1, glucose tolerance test (GTT) evidenced glucose intolerance with a 92% higher area under the curve of the glycemic response in HFHSD-fed mice compared to SD mice (p < 0.0001, Table 1, n = 12 per group). In agreement, insulin tolerance test (ITT) highlighted systemic insulin resistance, with a 38% lower area over the curve of the glycemic response in HFHSD-fed animals compared to controls (p < 0.01, Table 1, n = 12 per group). Altogether, these results confirm that the 14-weeks HFHSD diet induced significant alterations in body composition and systemic glucose homeostasis in mice, in favor with a progression towards type 2 diabetes^17^.

**Table 1:** Physiological parameters of SD and HFHSD mice.

	Ctrl (± SD)	HFHSD (± SD)	p-value	% variation
Animal weight (g)	27.48 (± 1.90)	45.23 (± 2.0)	****	65%
Glycaemia (mg/dL)	130.0 (± 13.6)	177.5 (± 23.4)	****	36%
GTT (AUC)	15,383 (± 2,768)	29,506 (± 5,020)	****	92%
ITT (AOC)	2,028 (± 365)	1,251 (± 762)	**	− 38%
Liver weight (mg)	1,378 (± 83)	2,166 (± 456)	****	57%
Lung weight (mg)	281 (± 20)	278 (± 20)	ns	1%

Results are expressed as mean ± standard deviation (SD). Data are mean of n = 12 mice per group for total weight, glycaemia, GTT and ITT (glucose and insulin resistance test respectively) and n = 8 mice per group for liver and lung weight; Results of unpaired two-tailed t-test are indicated as non-significant (ns), p < 0.01 (**) and p < 0.0001 (****). Percentages of variation between HFHSD and control fed mice are indicated.

### NMR determination of organ and serum metabolic profiles

We analyzed the metabolome of the lung by NMR spectroscopy and compared it with the metabolome of serum and five other organs (liver, heart, skeletal muscle, kidneys and brain) in both SD and HFHSD mice (n = 8 per group). This approach allowed the absolute quantification of several metabolites, allowing an inter-organ comparison of HFHSD-induced metabolic alterations. All serum and organ extracts delivered interpretable NMR spectra that displayed sharp peaks typical of small molecules, overlaid with broad signals originating mostly from lipids in organ extracts, or lipids plus proteins in serum. The careful analysis of the ^1^H 1D and the two-dimensional ^1^H–^1^H and ^1^H–^13^C NMR led to the identification and quantification of 44 metabolites in organs and 32 metabolites in serum. The relative lipid content could be also evaluated^18^ and results were confirmed by triglyceride (TG) biochemical quantification (see below).

#### Obesity-associated metabolic signature of the lung

**Figure 1:**
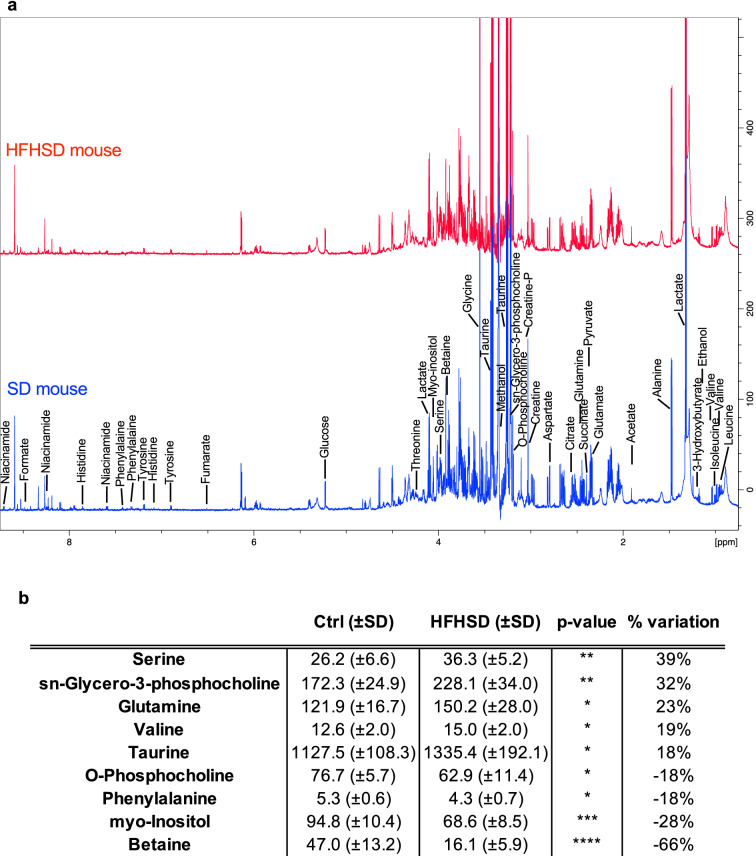
Metabolite modifications identified by NMR in the lung of HFHSD mice. (**a**) ^1^H-NMR representative spectra from lung from SD (blue) and HFHSD (red) fed mice. (**b**) List of metabolites differentially found in the lung of HFHSD mice compared to SD mice. Results are expressed as mean ± standard deviation (SD) and are in nmol/100 mg of lung. Data are mean of n = 8 mice per group; Results of unpaired two-tailed t-test are indicated p < 0.05 (*), p < 0.01 (**), p < 0.001 (***) and p < 0.0001 (****). Percentages of variation between HFHSD and SD fed mice are indicated. All metabolites quantified including non-significant variation are reported in Table 2.

**Figure 2:**
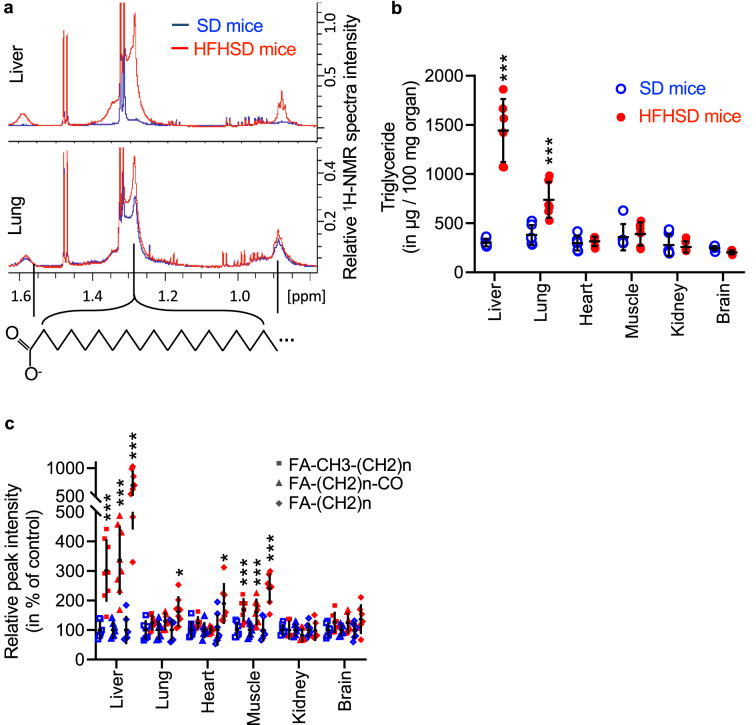
Lipids and specifically triglycerides accumulation in the lung in comparison with the liver or all other organs of SD and HFHSD mice. (**a**) ^1^H-NMR representative spectra from the lung (at bottom) and the liver (at top) from SD (blue) and HFHSD (red) fed mice. Spectra zoom highlights fatty acid (FA) areas of –C**H**_**2**_–CO (1.6 ppm), (C**H**_2_)n (1.3 ppm) and CH_2_–C**H**_3_ moieties (0.9 ppm). (**b**) Relative abundance of each typical lipid moieties –C**H**_**2**_–CO (triangle), (C**H**_2_)n (diamond) and CH_2_–C**H**_3_ (square) of SD (blue empty) or HFHSD (red full) fed mice estimated by integration of the ^1^H-NMR spectra. Data are mean of n = 8 mice per group; Results of unpaired two-tailed t-test are indicated as non-significant (NS) p < 0.05 (*), p < 0.01 (**), p < 0.001 (***) and p < 0.0001 (****). (**c**) Triglyceride contents in liver, lungs, heart, gastrocnemius muscles, kidneys and brain. Data are mean of n = 8 mice per group; Results of unpaired two-tailed t-test are indicated as non-determined (ND), non-significant (NS), p < 0.05 (*), p < 0.01 (**), p < 0.001 (***) and p < 0.0001 (****). (The quantification is also reported in μg/mg of total protein in Supplementary Fig. 2).

Among the 44 metabolites identified in all organs, 32 metabolites have been quantified in lung extracts (Table 2, n = 8 per group). NMR analysis of the lung showed that HFHSD induced significant changes in 9 metabolites compared to SD mice (Fig. 1). Betaine (trimethylglycine) concentration decreased by 66%, from 47 ± 13.2 nmol/100 mg of tissue to 16.1 ± 5.9 nmol/100 mg (p < 0.0001) while serine was increased by 38%, from 26.2 ± 6.6 nmol/100 mg to 36.3 ± 5.2 nmol/100 mg (p < 0.01). Sn-glycero-3-phosphocholine, glutamine, valine and taurine increased by 32% (p < 0.01), 23% (p < 0.05), 19% (p < 0.05), 18% (p < 0.05) respectively, while myo-inositol, phenylalanine and o-phosphocholine were decreased by 28% (p < 0.001), 18% (p < 0.05) and 18% (p < 0.05) respectively, in HFHSD lung compared to SD lung (Fig. 1b). Interestingly, we noted the presence of broad peaks on lung NMR spectra, that could be assigned to the CH3, CH2 and CH2-CO moieties of heterogeneous lipid species^18^. Their peak area was illustrated and quantified in Fig. 2 (panel a and b, respectively). To confirm these results, we decided to measure biochemically TG levels in the lung of SD and HFHSD mice (Fig. 2c). TG levels were 94% higher in lungs of HFHSD-fed animals compared to SD controls, averaging 736 ± 181 μg/100 mg and 380 ± 101 μg/100 mg respectively (p < 0.001), confirming lipid accumulation in the lung of HFHSD mice.

**Table 2:** Metabolite concentrations measured by NMR in the lung of SD and HFHSD mice.

	Lung (in nmol/100 mg)			
	Ctrl (± SD)	HFHSD (± SD)	p-value	% variation
2-Hydroxybutyrate	ND	ND		
3-Hydroxybutyrate	3.2 (± 2.5)	3.5 (± 1.8)	ns	8%
4-Aminobutyrate	ND	ND		
Acetate	7.7 (± 1.5)	7.8 (± 1.0)	ns	0%
Acetone	ND	ND		
Alanine	105.7 (± 12.0)	122.7 (± 18.2)	ns	16%
Aspartate	120.7 (± 23.8)	142.0 (± 27.0)	ns	18%
Betaine	47.0 (± 13.2)	16.1 (± 5.9)	****	− 66%
Carnosine	ND	ND		
Choline	25.0 (± 12.0)	24.1 (± 4.1)	ns	− 3%
Citrate	40.0 (± 5.7)	47.2 (± 9.9)	ns	18%
Creatine	46.0 (± 11.7)	45.9 (± 14.2)	ns	0%
Creatine-P	9.1 (± 3.4)	14.0 (± 3.2)	ns	54%
Dimethylglycine	ND	ND		
Ethanol	14.3 (± 3.2)	264.1 (± 509.8)	ns	1750%
Formate	2.5 (± 0.6)	3.3 (± 0.8)	ns	34%
Fumarate	1.5 (± 0.5)	1.5 (± 0.4)	ns	3%
Glucose	230.6 (± 44.0)	266.0 (± 91.4)	ns	15%
Glucose-1-P	ND	ND		
Glucose-6-P	ND	ND		
Glutamate	227.6 (± 19.9)	257.7 (± 49.5)	ns	13%
Glutamine	121.9 (± 16.7)	150.2 (± 28.0)	*	23%
Glutathione	ND	ND		
Glycine	391.5 (± 48.5)	359.8 (± 56.3)	ns	− 8%
Histidine	4.8 (± 1.0)	5.0 (± 1.0)	ns	3%
Isoleucine	6.0 (± 0.7)	6.3 (± 0.6)	ns	5%
Lactate	445.5 (± 65.4)	463.2 (± 115.7)	ns	4%
Leucine	11.6 (± 1.4)	10.6 (± 1.5)	ns	− 9%
Malonate	ND	ND		
Mannose	ND	ND		
myo-Inositol	94.8 (± 10.4)	68.6 (± 8.5)	***	− 28%
Niacinamide	14.1 (± 2.7)	12.2 (± 2.1)	ns	− 14%
O-Phosphocholine	76.7 (± 5.7)	62.9 (± 11.4)	*	− 18%
Pantothenate	ND	ND		
Phenylalanine	5.3 (± 0.6)	4.3 (± 0.7)	*	− 18%
Pyruvate	3.2 (± 1.2)	2.8 (± 1.0)	ns	− 12%
Sarcosine	ND	ND		
Serine	26.2 (± 6.6)	36.3 (± 5.2)	**	39%
sn-Glycero-3-phosphocholine	172.3 (± 24.9)	228.1 (± 34.0)	**	32%
Succinate	7.3 (± 2.2)	7.3 (± 1.8)	ns	1%
Taurine	1127.5 (± 108.3)	1335.4 (± 192.1)	*	18%
Threonine	30.5 (± 13.7)	27.5 (± 6.1)	ns	− 10%
Tyrosine	6.7 (± 1.6)	6.0 (± 0.8)	ns	− 11%
Valine	12.6 (± 2.0)	15.0 (± 2.0)	*	19%

Results are expressed as mean ± standard deviation (SD) and are in nmol/100 mg of lung. Data are mean of n = 8 mice per group; Results of unpaired two-tailed t-test are indicated as non-determined (ND), non-significant (NS), p < 0.05 (*), p < 0.01 (**), p < 0.001 (***) and p < 0.0001 (****). Percentages of variation between HFHSD and control fed mice are indicated.

#### Obesity-associated metabolic signatures in serum and other organs

NMR analyses of serum showed that glucose concentration increased by 31% in HFHSD mice compared to SD mice, varying from 3.82 ± 1.16 to 5.01 ± 0.55 mM (p < 0.02, Table 3, n = 8 per group), in agreement with the glycemia measurement using a glucometer (Table 1). NMR analyses also evidenced lower serum concentrations for allantoin, creatine and phenylalanine, and higher concentrations for alanine and 3-hydroxybutyrate in HFHSD-fed mice compared to controls (p < 0.05, Table 3). Citrate and succinate concentrations, two TCA-derived metabolites, were 28% and 32% higher in the serum of HFHSD-fed mice compared to controls, respectively (p < 0.02). Inversely, glycine, dimethylglycine (DMG) and betaine that derive from the 1C cycle metabolism were 34%, 68% and 54% lower in the serum of HFHSD-fed mice compared to controls, respectively (p < 0.01). No significant alteration of branched-chain amino acid concentrations was observed in serum (Table 3).

**Table 3:** Metabolite concentrations measured by NMR in the serum of SD and HFHSD mice.

	Serum metabolite (in microM)			
	Ctrl (± SD)	HFHSD (± SD)	p-value	% variation
3-Hydroxybutyrate	47.7 (± 9.9)	82.4 (± 24.9)	**	73%
Acetate	223.9 (± 64.4)	206.3 (± 30.8)	ns	− 8%
Alanine	156.8 (± 32.6)	218.0 (± 50.2)	*	39%
Allantoin	35.7 (± 8.2)	26.2 (± 8.1)	*	− 27%
Asparagine	22.0 (± 4.9)	25.4 (± 7.1)	ns	15%
Betaine	53.6 (± 8.3)	24.6 (± 10.7)	****	− 54%
Citrate	169.3 (± 19.7)	216.2 (± 17.5)	***	28%
Creatine	69.7 (± 7.7)	55.5 (± 8.9)	**	− 20%
Dimethylglycine	4.6 (± 0.6)	1.5 (± 0.3)	****	− 68%
Ethanol	308.4 (± 34.4)	266.7 (± 40.2)	*	− 14%
Formate	722.8 (± 257.6)	559.9 (± 56.1)	ns	− 23%
Fumarate	4.3 (± 1.3)	2.6 (± 0.7)	**	− 40%
Glucose	3816.8 (± 555.4)	5013.1 (± 1165.0)	*	31%
Glutamine	278.6 (± 26.0)	303.7 (± 40.2)	ns	9%
Glycine	147.3 (± 16.5)	97.5 (± 31.2)	**	− 34%
Histidine	38.1 (± 4.7)	36.2 (± 7.3)	ns	− 5%
Isobutyrate	5.6 (± 1.4)	5.8 (± 1.7)	ns	4%
Isoleucine	34.5 (± 8.9)	45.4 (± 13.4)	ns	32%
Lactate	2563.4 (± 504.8)	2336.6 (± 707.9)	ns	− 9%
Leucine	105.4 (± 16.8)	89.0 (± 19.5)	ns	− 16%
Lysine	139.5 (± 19.7)	135.5 (± 36.5)	ns	− 3%
Mannose	19.9 (± 7.5)	30.3 (± 12.1)	ns	52%
Methanol	120.9 (± 32.6)	149.1 (± 18.2)	ns	23%
Methionine	51.9 (± 9.5)	51.7 (± 8.7)	ns	0%
myo-Inositol	38.1 (± 3.6)	19.8 (± 4.1)	****	− 48%
Phenylalanine	33.5 (± 3.4)	27.1 (± 5.6)	*	− 19%
Pyruvate	62.9 (± 18.4)	65.4 (± 17.9)	ns	4%
Serine	86.8 (± 10.3)	84.1 (± 13.0)	ns	− 3%
Succinate	26.5 (± 8.1)	34.9 (± 2.7)	*	32%
Threonine	119.3 (± 17.5)	117.4 (± 14.8)	ns	− 2%
Tyrosine	70.5 (± 11.3)	64.0 (± 13.4)	ns	− 9%
Valine	119.1 (± 12.1)	139.2 (± 27.8)	ns	17%

Results are expressed as mean ± standard deviation (SD) and are in micromolar in serum. Data are mean of n = 8 mice per group; Results of unpaired two-tailed t-test are indicated as non-determined (ND), non-significant (NS), p < 0.05 (*), p < 0.01 (**), p < 0.001 (***) and p < 0.0001 (****). Percentages of variation between HFHSD and control fed mice are indicated.

The HFHSD had a significant influence and displayed tissue-specific profiles. Among the 44 quantified metabolites, 16, 8, 8 and 7 were significantly altered in the liver, heart, gastrocnemius muscle and kidneys, respectively. Interestingly, the less impacted organ was the brain which displayed only 2 altered metabolites following HFHSD (Tables 4, 5, 6, 7, 8, n = 8 per group).

**Table 4:** Metabolite concentrations measured by NMR in the liver of SD and HFHSD mice.

	Liver (in nmol/100 mg)			
	Ctrl (± SD)	HFHSD (± SD)	p-value	% variation
2-Hydroxybutyrate	4,8 (± 1.5)	172.4 (± 61.4)	****	3499%
3-Hydroxybutyrate	14.4 (± 2.1)	22.7 (± 9.2)	*	57%
4-Aminobutyrate	ND	ND		
Acetate	40.8 (± 14.1)	36.0 (± 10.1)	ns	− 12%
Acetone	ND	ND		
Alanine	423.6 (± 112.0)	373.8 (± 87.7)	ns	− 12%
Aspartate	28.8 (± 6.5)	25.4 (± 5.5)	ns	− 12%
Betaine	364.4 (± 129.6)	26.4 (± 14.3)	****	− 93%
Carnosine	ND	ND		
Choline	10.9 (± 3.6)	10.6 (± 2.5)	ns	− 3%
Citrate	50.0 (± 8.3)	54.4 (± 11.2)	ns	9%
Creatine	24.0 (± 4.2)	15.3 (± 3.0)	***	− 36%
Creatine-P	ND	ND		
Dimethylglycine	16.5 (± 4.7)	2.6 (± 0.6)	****	− 84%
Ethanol	38.5 (± 30.1)	26.7 (± 26.0)	ns	− 31%
Formate	3.2 (± 2.0)	3.5 (± 1.0)	ns	12%
Fumarate	4.1 (± 1.3)	4.9 (± 1.2)	ns	19%
Glucose	3221.2 (± 579.8)	4131.7 (± 758.9)	*	28%
Glucose-1-P	ND	ND		
Glucose-6-P	ND	ND		
Glutamate	129.8 (± 34.7)	185.7 (± 46.4)	*	43%
Glutamine	363.8 (± 105.9)	471.0 (± 90.5)	*	29%
Glutathione	788.9 (± 166.5)	733.9 (± 211.9)	ns	− 7%
Glycine	226.6 (± 62.9)	141.3 (± 25.3)	**	− 38%
Histidine	35.7 (± 7.6)	30.5 (± 6.8)	ns	− 14%
Isoleucine	20.3 (± 5.8)	16.6 (± 3.0)	ns	− 18%
Lactate	1249.0 (± 232.7)	1099.7 (± 219.3)	ns	− 12%
Leucine	38.0 (± 7.9)	27.4 (± 4.1)	**	− 28%
Malonate	ND	ND		
Mannose	21.8 (± 8.1)	23.2 (± 5.6)	ns	6%
myo-Inositol	34.1 (± 6.6)	11.1 (± 0.9)	**	− 67%
Niacinamide	16.1 (± 6.0)	17.0 (± 3.5)	ns	6%
*O*-Phosphocholine	98.5 (± 18.7)	78.3 (± 20.9)	ns	− 21%
Pantothenate	ND	ND		
Phenylalanine	11.2 (± 2.5)	8.4 (± 1.5)	*	− 25%
Pyruvate	6.4 (± 3.2)	8.1 (± 3.0)	ns	27%
Sarcosine	14.4 (± 5.2)	5.8 (± 2.8)	**	− 60%
Serine	ND	ND		
sn-Glycero-3-phosphocholine	73.2 (± 20.9)	61.8 (± 14.9)	ns	− 16%
Succinate	150.5 (± 53.7)	135.5 (± 30.7)	ns	− 10%
Taurine	699.6 (± 404.3)	2009.2 (± 388.0)	****	187%
Threonine	47.4 (± 14.1)	31.2 (± 5.7)	**	− 34%
Tyrosine	9.6 (± 1.8)	7.1 (± 1.4)	**	− 26%
Valine	34.1 (± 8.5)	27.7 (± 4.4)	ns	− 19%

Results are expressed as mean ± standard deviation (SD) and are in nmol/100 mg of liver. Data are mean of n = 8 mice per group; Results of unpaired two-tailed t-test are indicated as non-determined (ND), non-significant (NS), p < 0.05 (*), p < 0.01 (**), p < 0.001 (***) and p < 0.0001 (****). Percentages of variation between HFHSD and control fed mice are indicated.

**Table 5:** Metabolite concentrations measured by NMR in the heart of SD and HFHSD mice.

	Heart (in nmol/100 mg)			
	Ctrl (± SD)	HFHSD (± SD)	p-value	% variation
2-Hydroxybutyrate	ND	ND		
3-Hydroxybutyrate	6.9 (± 1.8)	7.6 (± 2.7)	ns	11%
4-Aminobutyrate	ND	ND		
Acetate	6.7 (± 1.2)	6.0 (± 1.4)	ns	− 11%
Acetone	ND	ND		
Alanine	264.3 (± 35.8)	363.2 (± 65.8)	**	37%
Aspartate	116.1 (± 21.9)	116.1 (± 22.9)	ns	0%
Betaine	ND	ND		
Carnosine	ND	ND		
Choline	10.6 (± 2.3)	8.7 (± 1.9)	ns	− 17%
Citrate	18.5 (± 7.3)	21.2 (± 4.5)	ns	15%
Creatine	1166.1 (± 185.1)	946.8 (± 132.4)	*	− 19%
Creatine-P	ND	ND		
Dimethylglycine	ND	ND		
Formate	8.4 (± 2.7)	18.0 (± 5.9)	**	114%
Fumarate	5.0 (± 1.3)	3.9 (± 1.2)	ns	− 22%
Ethanol	6.7 (± 2.0)	5.7 (± 1.1)	ns	− 14%
Glucose	38.3 (± 15.9)	61.8 (± 15.6)	*	61%
Glucose-1-P	ND	ND		
Glucose-6-P	ND	ND		
Glutamate	503.1 (± 103.4)	519.7 (± 57.6)	ns	3%
Glutamine	616.8 (± 161.1)	531.7 (± 81.1)	ns	− 14%
Glutathione	67.7 (± 20.9)	48.9 (± 13.8)	ns	− 28%
Glycine	60.4 (± 9.2)	57.9 (± 21.7)	ns	− 4%
Histidine	19.5 (± 2.8)	18.7 (± 2.8)	ns	− 4%
Isoleucine	7.1 (± 1.7)	9.0 (± 1.3)	*	27%
Lactate	1847.7 (± 455.5)	1717.2 (± 347.0)	ns	− 7%
Leucine	15.3 (± 1.7)	14.2 (± 2.3)	ns	− 7%
Malonate	23.2 (± 5.1)	19.5 (± 2.3)	ns	− 16%
Mannose	ND	ND		
Niacinamide	29.4 (± 9.8)	22.4 (± 7.6)	ns	− 24%
*O*-Phosphocholine	28.0 (± 4.0)	19.2 (± 2.7)	***	− 31%
myo-Inositol	25.4 (± 7.1)	21.4 (± 2.6)	ns	− 16%
Pantothenate	4.4 (± 0.9)	4.0 (± 0.7)	ns	− 8%
Phenylalanine	8.5 (± 0.9)	6.9 (± 1.2)	**	− 19%
Pyruvate	5.0 (± 1.1)	5.7 (± 1.7)	ns	14%
Sarcosine	ND	ND		
Serine	46.0 (± 11.4)	38.3 (± 8.1)	ns	− 17%
sn-Glycero-3-phosphocholine	27.6 (± 2.8)	24.4 (± 3.7)	ns	− 12%
Succinate	183.0 (± 52.3)	208.6 (± 35.1)	ns	14%
Taurine	4058.7 (± 844.1)	3563.8 (± 466.6)	ns	− 12%
Threonine	43.4 (± 11.2)	51.6 (± 15.7)	ns	19%
Tyrosine	9.4 (± 1.4)	8.5 (± 1.2)	ns	− 10%
Valine	12.8 (± 1.5)	17.2 (± 2.5)	**	34%

Results are expressed as mean ± standard deviation (SD) and are in nmol/100 mg of heart. Data are mean of n = 8 mice per group; Results of unpaired two-tailed t-test are indicated as non-determined (ND), non-significant (NS), p < 0.05 (*), p < 0.01 (**), p < 0.001 (***) and p < 0.0001 (****). Percentages of variation between HFHSD and control fed mice are indicated.

**Table 6:** Metabolite concentrations measured by NMR in the gastrocnemius muscle of SD and HFHSD mice.

	Muscle (in nmol/100 mg)			
	Ctrl (± SD)	HFHSD (± SD)	p-value	% variation
2-Hydroxybutyrate	ND	ND		
3-Hydroxybutyrate	7.9 (± 1.9)	9.5 (± 4.3)	ns	19%
4-Aminobutyrate	ND	ND		
Acetate	13.0 (± 2.5)	13.5 (± 3.8)	ns	4%
Acetone	ND	ND		
Alanine	466.3 (± 75.9)	601.2 (± 121.1)	*	29%
Aspartate	28.8 (± 11.7)	20.8 (± 6.5)	ns	− 28%
Betaine	ND	ND		
Carnosine	548.2 (± 52.7)	411.2 (± 93.7)	**	− 25%
Choline	ND	ND		
Citrate	25.8 (± 5.2)	26.3 (± 9.1)	ns	2%
Creatine	3797.6 (± 575.0)	4027.7 (± 614.1)	ns	6%
Creatine-P	331.9 (± 181.6)	309.9 (± 169.1)	ns	− 7%
Dimethylglycine	1.2 (± 0.4)	0.6 (± 0.1)	**	− 50%
Ethanol	88.8 (± 37.2)	121.6 (± 45.4)	ns	37%
Formate	4.4 (± 2.0)	5.9 (± 2.0)	ns	33%
Fumarate	2.8 (± 1.4)	2.8 (± 1.3)	ns	3%
Glucose	279.6 (± 87.6)	209.7 (± 82.5)	ns	− 25%
Glucose-1-P	43.6 (± 24.6)	22.2 (± 6.1)	*	− 49%
Glucose-6-P	339.3 (± 131.4)	225.8 (± 48.5)	ns	− 33%
Glutamate	85.5 (± 22.1)	75.6 (± 17.0)	ns	− 12%
Glutamine	309.0 (± 44.1)	298.1 (± 39.2)	ns	− 4%
Glutathione	121.3 (± 24.4)	97.0 (± 30.3)	ns	− 20%
Glycine	425.8 (± 59.2)	299.3 (± 64.7)	**	− 30%
Histidine	17..1 (± 3.9)	17.0 (± 3.5)	ns	− 1%
Isoleucine	14.7 (± 3.3)	16.9 (± 4.5)	ns	15%
Lactate	5105.2 (± 1923.0)	4418.1 (± 908.0)	ns	− 13%
Leucine	27.2 (± 5.6)	25.2 (± 6.7)	ns	− 7%
Malonate	73.9 (± 11.9)	74.0 (± 13.6)	ns	0%
Mannose	71.5 (± 32.4)	51.2 (± 15.0)	ns	− 28%
myo-Inositol	27.6 (± 6.4)	18.4 (± 6.4)	*	− 33%
Niacinamide	9.9 (± 2.7)	9.6 (± 2.8)	ns	− 4%
*O*-Phosphocholine	ND	ND		
Pantothenate	ND	ND		
Phenylalanine	15.1 (± 2.6)	13.9 (± 3.0)	ns	− 8%
Pyruvate	5.4 (± 1.3)	3.2 (± 1.6)	**	− 42%
Sarcosine	23.8 (± 5.5)	25.7 (± 3.9)	ns	8%
Serine	61.7 (± 15.9)	78.1 (± 21.3)	ns	27%
sn-Glycero-3-phosphocholine	ND	ND		
Succinate	121.5 (± 27.6)	124.5 (± 23.1)	ns	2%
Taurine	6922.4 (± 1688.5)	7818.7 (± 1281.3)	ns	13%
Threonine	124.0 (± 36.9)	107.8 (± 35.1)	ns	− 13%
Tyrosine	19.4 (± 3.3)	19.6 (± 4.5)	ns	1%
Valine	30.7 (± 7.4)	41.2 (± 8.2)	*	34%

Results are expressed as mean ± standard deviation (SD) and are in nmol/100 mg of gastrocnemius muscle. Data are mean of n = 8 mice per group; Results of unpaired two-tailed t-test are indicated as non-determined (ND), non-significant (NS), p < 0.05 (*), p < 0.01 (**), p < 0.001 (***) and p < 0.0001 (****). Percentages of variation between HFHSD and control fed mice are indicated.

**Table 7:** Metabolite concentrations measured by NMR in the kidney of SD and HFHSD mice.

	Kidney (in nmol/100 mg)			
	Ctrl (± SD)	HFHSD (± SD)	p-value	% variation
2-Hydroxybutyrate	ND	ND		
3-Hydroxybutyrate	16.2 (± 4.0)	21.2 (± 6.7)	ns	31%
4-Aminobutyrate	ND	ND		
Acetate	14.1 (± 3.4)	16.1 (± 5.2)	ns	14%
Acetone	4.9 (± 1.0)	3.5 (± 1.1)	*	− 28%
Alanine	165.0 (± 33.5)	241.3 (± 54.6)	**	46%
Aspartate	307.6 (± 72.2)	346.5 (± 101.5)	ns	13%
Betaine	282.8 (± 66.9)	141.8 (± 21.0)	***	− 50%
Carnosine	ND	ND		
Choline	190.4 (± 93.7)	214.2 (± 108.8)	ns	12%
Citrate	15.3 (± 6.7)	5.3 (± 2.1)	**	− 66%
Creatine	152.9 (± 38.5)	136.9 (± 34.0)	ns	− 10%
Creatine-P	ND	ND		
Dimethylglycine	ND	ND		
Ethanol	35.8 (± 12.1)	277.8 (± 436.5)	ns	676%
Formate	5.3 (± 0.9)	6.4 (± 1.3)	ns	21%
Fumarate	7.1 (± 4.0)	8.6 (± 2.4)	ns	20%
Glucose	473.6 (± 130.0)	714.7 (± 209.3)	*	51%
Glucose-1-P	ND	ND		
Glucose-6-P	ND	ND		
Glutamate	1329.5 (± 323.6)	1368.0 (± 267.3)	ns	3%
Glutamine	208.6 (± 43.4)	231.2 (± 39.0)	ns	11%
Glutathione	ND	ND		
Glycine	843.1 (± 235.5)	782.1 (± 149.1)	ns	− 7%
Histidine	21.8 (± 4.7)	20.0 (± 4.3)	ns	− 8%
Isoleucine	22.1 (± 3.3)	26.3 (± 5.0)	ns	19%
Lactate	1236.3 (± 278.8)	1374.2 (± 286.1)	ns	11%
Leucine	47.1 (± 10.6)	45.8 (± 11.0)	ns	− 3%
Malonate	ND	ND		
Mannose	6.5 (± 2.5)	4.1 (± 2.8)	ns	− 38%
myo-Inositol	1337.3 (± 259.1)	1208.7 (± 232.1)	ns	− 10%
Niacinamide	25.8 (± 11.6)	26.5 (± 7.8)	ns	3%
*O*-Phosphocholine	313.5 (± 52.7)	204.2 (± 42.6)	**	− 35%
Pantothenate	3.4 (± 1.0)	3.7 (± 0.8)	ns	7%
Phenylalanine	16.7 (± 3.2)	16.0 (± 4.7)	ns	− 4%
Pyruvate	11.6 (± 7.1)	7.5 (± 1.9)	ns	− 35%
Sarcosine	ND	ND		
Serine	69.1 (± 9.9)	71.8 (± 21.5)	ns	4%
sn-Glycero-3-phosphocholine	1233.4 (± 291.3)	1605.3 (± 213.4)	*	30%
Succinate	182.0 (± 55.5)	216.0 (± 55.1)	ns	19%
Taurine	2485.9 (± 448.1)	2790.3 (± 419.1)	ns	12%
Threonine	69.4 (± 14.5)	70.5 (± 12.5)	ns	2%
Tyrosine	19.1 (± 5.8)	21.1 (± 6.0)	ns	10%
Valine	38.7 (± 9.1)	47.5 (± 12.3)	ns	23%

Results are expressed as mean ± standard deviation (SD) and are in nmol/100 mg of kidney. Data are mean of n = 8 mice per group; Results of unpaired two-tailed t-test are indicated as non-determined (ND), non-significant (NS), p < 0.05 (*), p < 0.01 (**), p < 0.001 (***) and p < 0.0001 (****). Percentages of variation between HFHSD and control fed mice are indicated.

**Table 8:** Metabolite concentrations measured by NMR in the brain of SD and HFHSD mice.

	Brain (in nmol/100 mg)			
	Ctrl (± SD)	HFHSD (± SD)	p-value	% variation
2-Hydroxybutyrate	ND	ND		
3-Hydroxybutyrate	5.7 (± 1.6)	7.3 (± 3.8)	ns	29%
4-Aminobutyrate	559.5 (± 110.9)	506.2 (± 172.8)	ns	− 10%
Acetate	19.7 (± 3.2)	19.0 (± 4.1)	ns	− 4%
Acetone	ND	ND		
Alanine	104.2 (± 20.9)	124.8 (± 63.7)	ns	20%
Aspartate	281.5 (± 82.2)	260.0 (± 71.6)	ns	− 8%
Betaine	ND	ND		
Carnosine	17.7 (± 10.0)	12.7 (± 8.3)	ns	− 28%
Choline	35.7 (± 7.4)	29.5 (± 5.5)	ns	− 17%
Citrate	25.6 (± 6.7)	35.9 (± 17.3)	ns	40%
Creatine	1195.9 (± 294.8)	974.2 (± 225.5)	ns	− 19%
Creatine-P	ND	ND		
Dimethylglycine	0.6 (± 0.2)	0.9 (± 0.4)	ns	47%
Ethanol	12.5 (± 2.9)	314.3 (± 507.3)	ns	2420%
Formate	5.4 (± 0.9)	6.5 (± 1.4)	ns	19%
Fumarate	2.4 (± 1.0)	2.8 (± 0.7)	ns	17%
Glucose	ND	ND		
Glucose-1-P	ND	ND		
Glucose-6-P	ND	ND		
Glutamate	1610.2 (± 269.5)	1364.5 (± 212.4)	ns	− 15%
Glutamine	782.3 (± 240.6)	609.4 (± 123.3)	ns	− 22%
Glutathione	79.1 (± 28.4)	93.2 (± 96.5)	ns	18%
Glycine	158.1 (± 74.9)	146.5 (± 25.9)	ns	− 7%
Histidine	8.0 (± 1.8)	8.0 (± 5.4)	ns	0%
Isoleucine	4.5 (± 1.3)	6.6 (± 3.0)	ns	47%
Lactate	1618.1 (± 255.2)	1699.1 (± 221.6)	ns	5%
Leucine	10.8 (± 1.5)	11.7 (± 3.6)	ns	9%
Malonate	22.8 (± 7.9)	20.9 (± 4.8)	ns	− 8%
Mannose	ND	ND		
myo-Inositol	868.7 (± 186.7)	746.2 (± 190.6)	ns	− 14%
Niacinamide	8.1 (± 2.3)	7.5 (± 1.2)	ns	− 8%
O-Phosphocholine	101.7 (± 16.4)	89.5 (± 13.6)	ns	− 12%
Pantothenate	4.3 (± 1,4)	2.8 (± 0.9)	*	− 34%
Phenylalanine	7.4 (± 1.4)	6.6 (± 1.4)	ns	− 10%
Pyruvate	10.9 (± 6.1)	13.5 (± 4,8)	ns	24%
Sarcosine	ND	ND		
Serine	112.4 (± 19.7)	119.8 (± 31.0)	ns	7%
sn-Glycero-3-phosphocholine	176.0 (± 41.3)	163.1 (± 34.5)	ns	− 7%
Succinate	103.7 (± 29.6)	95.7 (± 19.9)	ns	− 8%
Taurine	1729.2 (± 374.3)	1642.9 (± 395.2)	ns	− 5%
Threonine	50.6 (± 10.0)	48.5 (± 15.6)	ns	− 4%
Tyrosine	8.8 (± 1.5)	7.6 (± 1.3)	ns	− 13%
Valine	10.0 (± 2.0)	15.3 (± 4.7)	*	53%

Results are expressed as mean ± standard deviation (SD) and are in nmol/100 mg of brain. Data are mean of n = 8 mice per group; Results of unpaired two-tailed t-test are indicated as non-determined (ND), non-significant (NS), p < 0.05 (*), p < 0.01 (**), p < 0.001 (***) and p < 0.0001 (****). Percentages of variation between HFHSD and control fed mice are indicated.

All the organs studied harbored one or several unique metabolite alterations which could constitute potential biomarker candidates of the organ physiology adaptation to diet. Indeed, pantothenate was 34% lower (p < 0.05) in the brain (Table 8); citrate and acetone were 66% (p < 0.01) and 28% (p < 0.05) respectively lower in kidney (Table 7); Glucose-1-p, pyruvate and carnosine were 49% (p < 0.05), 42% (p < 0.01) and 25% (p < 0.01) respectively lower in gastrocnemius muscle (Table 6); Ethanol and isoleucine increase were 114% (p < 0.01) and 27% (p < 0.05) while niacinamide decrease was 31% (p < 0.001) altogether only in heart (Table 5). Lastly, liver showed the most unique metabolite alterations with a notable concentration increase by 3,499% of 2-hydroxybutyrate from 4.8 ± 1.5 nmol/100 mg of tissue to 172.4 ± 61.4 nmol/100 mg (p < 0.0001). 3-hydroxybutyrate and glutamate were increased by 57% (p < 0.05) and 43% (p < 0.05) respectively while sarcosine, threonine, leucine and tyrosine were decreased by 60% (p < 0.01), 34% (p < 0.01), 28% (p < 0.01) and 26% (p < 0.01) respectively (Table 4).

**Figure 3:**
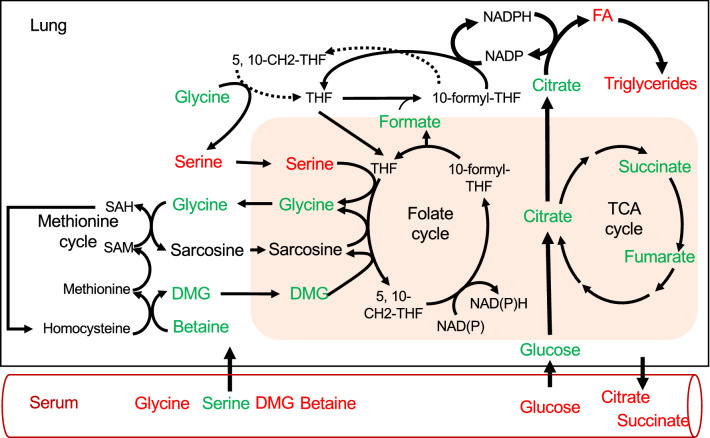
The accumulation of lipids is linked to alterations in the 1C cycle but not to the TCA pathway derived metabolites in the lung of HFHSD mice. Schematic diagram of 1C (methionine plus folate cycles) and TCA cycles, their subcellular compartmentations (mitochondrion in pink) and their potential link with de novo lipogenesis^30,31^. HFHSD causes significant modifications in 1C-derived metabolites in the lung and serum, and a significant modification in TCA-derived metabolites only in serum. Modified metabolites are represented in red, unmodified in green and non-detected in black. 1C-derived metabolites were also altered in other organ and specifically in the liver but were not represented here.

Some metabolite alterations are shared between two or more organs. For example, the HFHSD induced a significant rise of glucose concentration by 28%, 61% and 51% in the liver, heart and kidneys, respectively (p < 0.05, Tables 4, 5 and 7), but not in gastrocnemius muscle. Glucose was not detected in the brain, probably because of its rapid consumption before methanol metabolism quenching (Table 8). Interestingly, the levels of the TCA cycle-derived metabolites, such as citrate, succinate and fumarate, were not significantly altered in organs of HFHSD-fed animals compared to controls, except in kidneys where the citrate concentration was 61% lower (p < 0.01) (Table 7). Based on our results in lung and serum of HFHSD mice, we focused our analysis on the 1C pathway, with an emphasis on the methionine and folate cycles to highlight the central role of glycine and its N-methylated analogs that are shared by both cycles (Fig. 3). The liver evidenced the strongest decrease in the methionine cycle with 38% lower concentrations in glycine, 60% in sarcosine, 84% in DMG and 93% in betaine in HFHSD-fed mice compared to controls (p < 0.003, Table 4). Similarly, in gastrocnemius muscle, both glycine and DMG concentrations were 30% and 50% lower in HFHSD-fed mice compared to controls, respectively (p < 0.002, Table 6). In kidneys, betaine was 50% lower in HFHSD-fed mice compared to controls (p < 0.0003, Table 7), as observed above in the lung. Globally, all these observations evidenced important variations in the 1C pathway in several tissues of HFHSD-fed mice.

As observed for the lung, we noted the presence of broad peaks on NMR spectra of some tissues (Fig. 2a,b), that could be assigned to the CH_3_, CH_2_ and CH_2_-CO moieties of heterogeneous lipid species. We also quantified the TG levels in all organs. As showed on Fig. 2c, TG levels were 378% higher in the liver of HFHSD mice compared to SD mice, but unaltered in the other organs. The difference between NMR and triglycerides quantification in the skeletal muscles, and to a lesser extent in the heart, may indicate the accumulation of free fatty acids in these organs, which can be detected by NMR only, a phenomenon already observed with high-fat diets^19^.

#### Main comparison between metabolomes of lung, serum and other organs

Obesity-associated metabolome of the lung displayed specific metabolic alterations that are not found in serum and other organs, such as an increase by 36% (p < 0.01) in serine levels (Fig. 1b and Table 2). It should be noted that serine could not be determined in liver samples due to peak overlap with unidentified peaks on the NMR spectra. We therefore cannot exclude that this modification may be shared with the liver.

Yet, some metabolic signatures of the lung were shared with other organs. For example, a reduction in phenylalanine was found in the lung, serum and liver of HFHSD mice, whereas betaine was significantly decreased in the lung, serum, liver and kidney (Tables 2, 3, 4, 7). In agreement, other metabolites of the 1C pathway, such as glycine and DMG, were reduced in HFHSD mice in serum, liver and skeletal muscle (Tables 3, 4, 6), while sarcosine was specifically decreased in the liver of HFHSD compared to SD (Table 4). Altogether, these data point a strong reduction of 1C metabolism with obesity.

While the liver remained the most altered organ in HFHSD, it shared with the lung several metabolic features such as decreases in betaine, myo-inositol, o-phosphocholine and phenylalanine and increases in glutamine and taurine (Tables 2, 4). In addition, both tissues shared an accumulation of TG that was not found in other organs (Fig. 2). Finally, TCA metabolism showed organ-specific alterations in HFHSD mice. TCA cycle-derived metabolites were not impacted in the majority of organs including the lung but citrate and succinate concentrations were higher in the serum and citrate was reduced in kidneys of HFHSD mice compared to SD.

## Discussion

Obesity is associated with numerous organ dysfunctions, particularly in the lung with increased sensitivity to both chronic and acute illnesses^1–9,20–24^. To get insights into the contribution of cell metabolism alterations to lung fragility, we used NMR to compare the metabolic changes induced by obesity in the lung in comparison to serum and five organs (liver, heart, skeletal muscle, kidneys and brain). Our results demonstrate mainly that the lung displays both specific and shared metabolic changes following HFHSD, compared to serum and/or organs. The obesity-associated metabolic signature of the lung is nearest to the liver rather than other organs, sharing reduction in 1C metabolism and induction of lipid accumulation. Opposite, TCA-derived metabolites are not regulated in the lung and the majority of organs of HFHSD mice, whereas some of them are increased in serum (citrate and succinate) and reduced in kidneys (succinate). Altogether, our data point that obesity (i) mainly affects 1C metabolism in both serum and organs, including the lung, (ii) specifically regulates TCA metabolism in serum and kidney and (iii) specifically increases serine levels in the lung of HFHSD mice.

One important observation of our study is that HFHSD-fed mice presented serum- and organ-specific metabolomics adaptations. Care should be taken as some metabolic adaptations in organs may not only be a consequence of the diet. Indeed, obesity per se*,* rather than overnutrition, can induce organ-specific metabolic adaptations. This question was raised by Mora-Ortiz et al*.* who examined *db/db* mice organs^25^, a natural genetic model of obesity and also reported a decrease of 1C related metabolite in many organs (a glycine decrease in kidneys and distal colon; a homoserine decrease in liver and plasma; a serine decrease in brain, kidney and spleen). Unfortunately, they did not include lung in their study.

One striking observation is that HFHSD-feeding dampened 1C metabolism in all tested samples and specifically increased serine in the lung of HFHSD mice, whereas the TCA-derived metabolites were loosely regulated in several tissues, including the liver and the lung. In the liver, the importance of 1C cycle alterations with diet-induced obesity is supported by another multi-omics analyses^26^, whereas alterations in gene expression and metabolite abundances of TCA cycle were found in the liver of 27-week HFHSD-fed mice (60% [w/w] fat, 9.4% [w/w] sucrose)^27^. Interestingly, both 1C and TCA pathways could be interrelated and influence each other. Indeed, it is well accepted that under obesogenic conditions, the TCA-cycle is increased and produces citrate that provides carbon atoms for de novo fatty acid (FA) synthesis in the cytosol^28^. This anabolic pathway requires NADPH, which can be produced from different sources, such as the pentose phosphate pathway^29^. Recent studies suggest that the 1C pathway, and in particular serine, contributes in cytosolic NADPH production, as mutations that blocked mitochondrial folate metabolism resulted in fatty acid labeling from deuterated serine^30–32^. Therefore, although TCA-derived metabolites were not significantly modified by HFHSD in the lung, the increase in serine levels could sign an increase in de novo lipogenesis contributing to the accumulation of lipids observed in this organ. In agreement, increased serine levels was previously reported in the liver of diabetic rats which also have increased lipogenesis^33^. At the same time, we did not find clear modulation of the TCA-derived metabolites in the liver of HFHSD mice. Liver is known to highly respond to HFHSD and perform de novo lipogenesis^34^, that is fueled from TCA metabolites^28^. Therefore, our results suggest that metabolic fingerprints by NMR may not be able to detect modifications of de novo lipogenesis or TCA cycles. Those are rather detected in serum of HFHSD mice that reflects an homeostatic mechanism to regulate intracellular metabolite levels by secretion or exchange with biofluids (interstitial fluid, serum, urine)^35^. On the opposite, the concentration of metabolites derived from the 1C cycle was decreased in serum and in most organs, except in the heart and brain, indicating that the 1C pathway is highly sensitive and adaptable to the physiological environment and represent a relevant research perspective in the understanding of organ-specific metabolic alterations induced by obesity.

Lipid content is increased both in the liver and lung of the HFHSD mice, but not in the other organs considered in this study. Lipid accumulation in the liver is well documented, contrary to the lung. In the liver, lipid accumulation arises from both exogenous lipid storage and de novo lipogenesis using carbons derived from carbohydrates^34^. During a high-fat diet, exogenous fatty acids inhibit de novo lipogenesis and are mainly responsible for steatosis^36,37^. Accumulation of fatty acids in the lung is less understood. Lipid-targeted metabolomics studies by mass spectrometry showed significant quantitative and qualitative differences in neutral lipids, fatty acids, phospholipids and sphingolipids content in lung between HFHSD and very high fat diets^4^, while no lipid accumulation occurred in the lung with SD^38^. As in the liver, the lipid accumulation in the lung correlated with alterations in the levels of many metabolites, with almost 30% of the quantified metabolites affected. Nevertheless, the patterns of metabolic alterations in the lung and liver were distinct. Other lipidomics signature using coupled gas chromatography- mass spectrometry analyses have been reported in the lung following different obesogenic diets, which are dependent of the type of diets^4^, confirming lipotoxicity in the lung with obesity. As the lipotoxicity contributes to dysfunction and pathological states in organs, such as the hepatic lipid accumulation participates to nonalcoholic fatty liver diseases, an involvement of the lung lipid storage is possibly involved in obesity-associated complications such as asthma and COPD, or increased susceptibility to infectious diseases such as seasonal flu or SARS-CoV-2.

In conclusion, our results provide insights on the influence of HFHSD on mice's internal organ metabolism, especially and unexpectedly on the lung metabolism. Our analysis suggests that the TG deposition in the lungs, as in the liver but not in other organs, may be related to adaptations of the 1C metabolism. Mitochondrial metabolism may be central to the obesity-induced metabolic remodeling and its cellular outcomes.

## Methods

The study was carried out in compliance with the ARRIVE guidelines^39^. Mice were maintained and all experiments were performed in accordance the French guidelines for the care and use of animals^40^ and were approved by a regional ethic committee agreed by the French Ministry for Education and Research and set up by the agreed institution PBES (Ecole Normale Supérieure de Lyon) (APAFIS#4103-2016010417246403).

### Animal study

Male C57BL/6J mice (5-weeks-old, n = 12/group) (Envigo, France) were fed with a standard diet (SD, Rhod16A, GENOBIOS) or a high fat-high sucrose diet (HFHSD, 36% w/w fat, 16.6% w/w sucrose, Envigo, France) ad libitum for 14 weeks. Body weight was monitored weekly. Glucose and insulin tolerance tests (GTT and ITT respectively) were performed the last week of feeding on all mice and were performed with a delay of 4 days (meaning that GTT was performed at the beginning of the 13th week and ITT at the end of the week. For GTT and ITT, mice were fasted for 6 or 16 h respectively. Then, glucose (2 mg/g body weight) or insulin (0.75 mU/g body weight) were injected i.p. and blood glucose levels were monitored at the indicated time points. At the end of the feeding protocol, mice at fed state were anesthetized with isoflurane for retro-orbital blood functions and sacrificed by cervical dislocation for removing organs. Blood samples were kept on wet ice for a maximum of 1 h before being centrifuged at 5000*g* for 10 min in a 4 °C refrigerated centrifuge for serum removal. Aliquots of serum were stored at − 80 °C. Lungs, heart, gastrocnemius muscles, kidneys, brains were harvested, weighed, divided, snap-frozen in liquid nitrogen and stored at − 80 °C.

### Biochemical metabolite measurement

For ITT and GTT, glycemia was monitored every 15 min for 90 and 60 min respectively using an Accu-Chek (Roche, France) glucose meter. Organ triglyceride content was determined on total lysate of tissues according to the manufacturer’s instruction (Biolabo 87319).

### Metabolite extraction and sample preparation for NMR analyses

Metabolites were extracted using 100% methanol and a Precellys Homogeneiser according to the manufacturer's instructions. Full kidneys or 100 mg of all other frozen organs were put in 1 mL for muscles or 500 µL for other organs of 100% methanol and rapidly broken using Precellys homogenizer with ceramic beads (6 per tubes) and 6600 RPM twice 10 s with 10 s pause program at room temperature. The program was repeated again for muscles. The total lysate was put into 5 mL glass tubes and the Precellys tubes rinsed with 500 µL of 100% methanol. The glass tubes were hermetically closed and stored at − 20 °C. The extracts were dried under a gentle N2 flow until complete evaporation (approximately 10–12 h), then stored at − 20 °C until NMR sample preparation, right before analysis. 800 μL of D_2_O phosphate buffer prepared as described in Ref.^41^ were used to dissolve the dried lysate residues by vortexing for 30 s. Extracts were then transferred to 1.5 mL Eppendorf tubes and centrifuged at 13,000 rpm for 1 min at 4 °C. 550 μL of supernatant were transferred to 5 mm NMR tubes. 100 µL of serum were diluted with 100 µL of D_2_O phosphate buffer and directly transferred to 3 mm NMR tubes. Organ triglyceride content was determined on total lysate according to the manufacturer’s instructions (Biolabo 87319).

### NMR data acquisition and processing

All organ extracts and serum NMR spectra were obtained on a Bruker Avance III spectrometer operating at a 1H frequency of 800.14 MHz, equipped with a 5 mm TXI probe. The serum samples were measured on a Bruker 600 MHz NMR spectrometer equipped with a 5 mm TCI cryoprobe. All spectra were acquired at 303°K. The NMR samples were maintained at 4 °C before data acquisition and handled by a Bruker SampleJet high-throughput sample changer. Standard NOESY and CPMG with water presaturation ^1^H 1D NMR spectra were acquired on each sample, with 128 scans (512 for serum) and a spectral width of 20 ppm. For both sequences, the relaxation delay was set to 4 s. 2D NMR experiments (^1^H–^1^H TOCSY, ^1^H–^13^C HSQC and J-Resolved) were recorded with standard parameters^41^ on a subset of samples to achieve peaks assignment of the metabolites signal. All free induction decays (FIDs) were multiplied by an exponential function leading to a 0.3 Hz line-broadening factor before Fourier transform. ^1^H-NMR spectra were manually phased and referenced to the 5.230 glucose doublet using Topspin 3.6 (Bruker GmbH, Rheinstetten, Germany).

### NMR spectra analyses

The identification of the metabolites was carried out from the ^1^H 1D NMR data using the software ChenomX NMR Suite 8.0 (ChenomX Inc., Edmonton, Canada) and confirmed from analysis of 2D ^1^H–^1^H TOCSY, ^1^H–^13^C HSQC and J-Resolved NMR spectra. Metabolite concentrations were determined from the ^1^H 1D NOESY experiments using ChenomX software. A pure lactate solution (1 g/L, Fisher) was used as a concentration reference and exploited using the ERETIC2 utility from TopSpin to add a digitally synthesized peak to a spectrum^42^. The relative abundance of NMR observed lipids massifs were determined by global spectral integration: (**CH3–**, 0.86–0.91 ppm; –**CH2**–, 1.25–1.30 ppm; CH2–**CH2**–CO, 1.55–1.62 ppm; **CH2**–CO, 2.22–2.26 ppm)^18^.

### Statistics

All data were expressed as the mean ± SD. Statistical comparisons were performed using Student's t-test.

## Supplementary Information

Supplementary Information.

## Data Availability

The data used in this study are available upon request. NMR spectra were deposited at the Metabolight database (https://www.ebi.ac.uk/metabolights/MTBLS2521).

## Abbreviations

1C: One-carbon
COPD: Chronic obstructive pulmonary disease
DMG: Dimethylglycine
GTT: Glucose tolerance tests
HFHSD: High-fat and high-sucrose diet
ITT: Insulin tolerance test
NMR: Nuclear magnetic resonance
SD: Standard diet
SEM: Standard error of the mean
TCA: Tricarboxylic acid
TG: Triglycerides
