# Supplementary material for: Dietary obesity in mice is associated with lipid deposition and metabolic shifts in the lungs sharing features with the liver
Source: Sci Rep. 2021 Apr 22;11:8712. doi: 10.1038/s41598-021-88097-8 (PMC8062462; doi:10.1038/s41598-021-88097-8)

Supplementary figure 1: Glucose tolerance tests performed in SD and HFHSD mice

Curve representation of glycaemia following glucose injection in GTT assay performed after 13 weeks of SD or HFHSD diet. Means +/- SD.

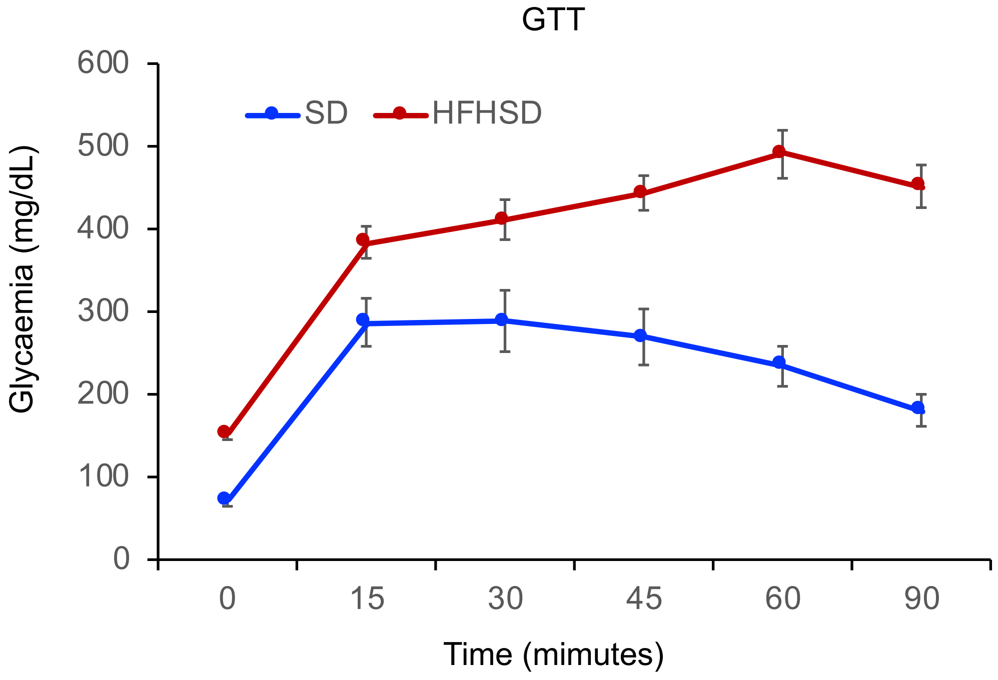

Supplementary figure 2: TG quantification in tissues of SD and HFHSD mice

Complementary to figure 1a, triglyceride (TG) quantification was normalized to the total protein content obtain by Bradford assay.

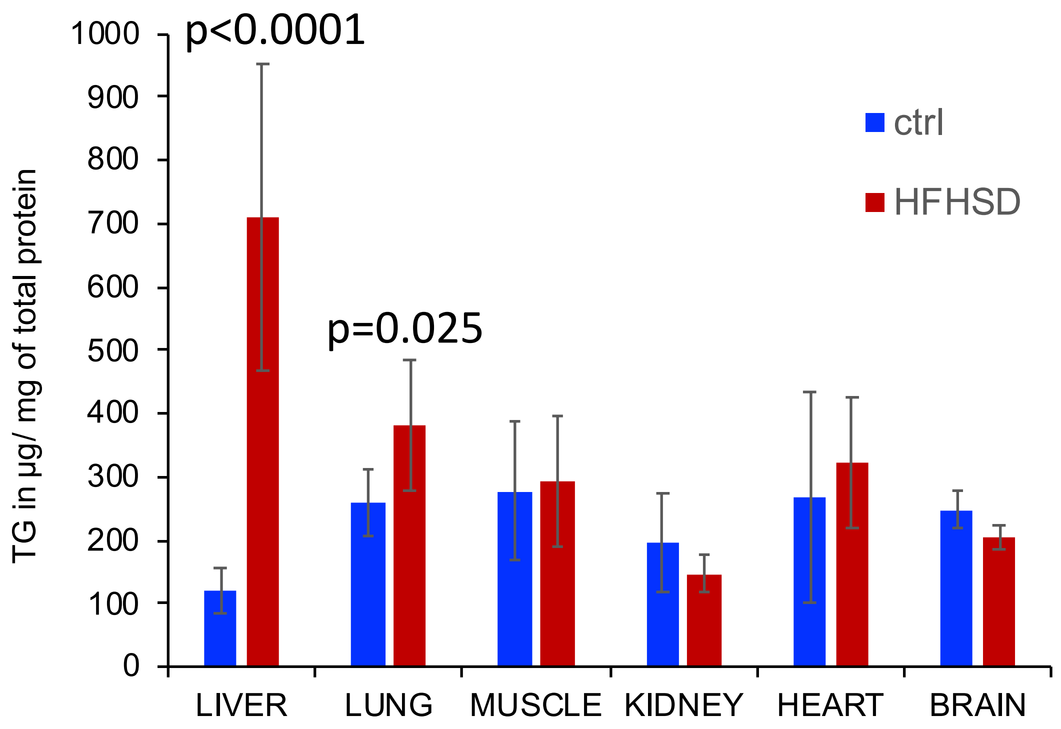

Supplement: Supplementary file 1 — Supplementary Information. [file 41598_2021_88097_MOESM1_ESM.pdf]
